# Case Report: Prolonged extracorporeal membrane oxygenation as a deliberate operative adjunct for tracheobronchial reconstruction in a child with invasive aspergillosis

**DOI:** 10.3389/fped.2026.1843892

**Published:** 2026-05-28

**Authors:** Marcin Łosin, Wojciech Karolak, Romuald Lango, Witold Rzyman, Monika Rosinski, Weronika Lotkowska, Piotr Czauderna

**Affiliations:** 1 Department of Surgery and Urology for Children and Adolescents, Medical University of Gdansk, Gdansk, Poland; 2 Department of Cardiac and Vascular Surgery, Faculty of Medicine, Medical University of Gdansk, Gdansk, Poland; 3 Department of Thoracic Surgery, Medical University of Gdansk, Gdansk, Poland; 4 Department of Cardiac and Vascular Surgery, University Clinical Center, Gdansk, Poland

**Keywords:** carinal reconstruction, esophageal flap, extracorporeal membrane oxygenation (ECMO), invasive aspergillosis infection, pediatric surgery, tracheobronchial reconstruction, tracheo-esophageal fistula (TEF)

## Abstract

We report the case of a 6-year-old boy with newly diagnosed type 1 diabetes mellitus presenting in diabetic ketoacidosis, who developed a massive acquired tracheo-esophageal fistula (TEF) with extensive destruction of the tracheobronchial anatomy, including near-complete loss of the left main bronchus, secondary to invasive mediastinal aspergillosis. The clinical course was complicated by refractory respiratory failure and subsequent right ventricular failure, necessitating prolonged extracorporeal membrane oxygenation (ECMO) support totaling 96 days. The patient required veno-venous ECMO, followed by temporary conversion to veno-arterial ECMO to stabilize hemodynamics during profound atelectasis and pulmonary hypertension, before returning to veno-venous support until decannulation. Definitive airway reconstruction was achieved using a vascularized autologous esophageal muscular flap in a severely inflamed and infected operative field where prosthetic materials were contraindicated, with planned sacrifice of esophageal continuity to prioritize airway restoration. Antifungal therapy was initiated following histopathological confirmation of Aspergillus hyphae and adjusted in response to hepatic dysfunction. Postoperatively, intentional lung rest for 14 days, with gas exchange maintained entirely by ECMO, protected the fragile membranous reconstruction. Despite multiple life-threatening complications, survival with satisfactory neurological outcome was achieved through close multidisciplinary cooperation. This case illustrates the role of ECMO as a bridge to complex airway reconstruction in pediatric patients with destructive invasive fungal disease, and describes the esophageal muscular wall flap as an autologous salvage option for posterior tracheobronchial defects in infected mediastinal fields.

## Introduction

Extracorporeal membrane oxygenation (ECMO) is increasingly used in pediatric intensive care, with improving outcomes driven by advances in technology, anticoagulation strategies, and growing institutional experience (1, 2). Although ECMO is most commonly employed for potentially reversible cardiac or respiratory failure of limited duration, prolonged support may be required in selected patients with complex surgical and infectious pathology (3).

Acquired tracheo-esophageal fistula (TEF) in children is rare and is typically associated with prolonged intubation, caustic ingestion, trauma, or malignancy (4, 5). TEF secondary to invasive fungal infection is exceptionally uncommon, particularly in immunocompetent children (6, 7). Reconstruction of large airway defects in an infected mediastinum presents a unique challenge: prosthetic materials are prone to colonization and failure, while suitable vascularized autologous tissue options are limited (8).

We describe a child with newly diagnosed type 1 diabetes mellitus who developed a massive acquired TEF with extensive tracheobronchial necrosis caused by invasive mediastinal aspergillosis, managed with prolonged ECMO support and autologous esophageal tissue reconstruction.

## Case report

### Presentation and initial investigations

A previously healthy 6-year-old German boy presented on August 4, 2023, to a regional hospital in Siberia while on holiday, with malaise and respiratory symptoms. He was diagnosed with diabetic ketoacidosis due to previously unrecognized type 1 diabetes mellitus. Contrast-enhanced chest computed tomography performed on August 8 demonstrated a posterior mediastinal mass compressing the left main bronchus and deforming the carina. Flexible bronchoscopy confirmed left bronchial compression and carinal flattening, while esophagogastroduodenoscopy was unremarkable. He was discharged after 10 days with advice to continue diagnostic evaluation in Germany.

During subsequent air travel to Kaliningrad, the patient developed paroxysmal coughing followed by frothy vomiting. His condition deteriorated rapidly, and he presented to an emergency department in severe respiratory distress, before transfer to the Provincial Integrated Hospital in Elblag, Poland.

### Emergency management and transfer

In the emergency department, sudden desaturation and bradycardia progressed to cardiac arrest requiring cardiopulmonary resuscitation. Following endotracheal intubation, marked gastric distension raised suspicion of an airway-esophageal communication. Emergency gastroscopy revealed esophageal perforation with a large TEF, and a covered esophageal stent (18 mm; smallest available adult size) was placed as a temporizing measure. The patient was transferred to the Pediatric Surgery and Intensive Care Unit at the University Clinical Center in Gdansk, Poland.

Repeat computed tomography no longer demonstrated the previously described mediastinal mass (Figures 3A,B). Endoscopic reassessment revealed a large TEF measuring approximately 2–3 cm at the level of the carina, with extensive destruction of the posterior tracheal wall and adjacent esophagus, just proximal to the left main bronchus. Despite maximal conventional ventilation, the patient developed progressive respiratory failure due to aspiration pneumonitis and inability to ventilate effectively through the airway defect. Veno-venous ECMO (VV-ECMO) was initiated on August 19, 2023, with ultrasound-guided percutaneous cannulation of the right internal jugular vein (17 Fr drainage cannula) and right femoral vein (23 Fr return cannula), confirmed by transthoracic echocardiography. A bolus of 2,500 IU of unfractionated heparin was administered at cannulation.

### Definitive surgical management

Despite removal of the esophageal stent, endoluminal vacuum therapy, and temporary right bronchial stenting, the defect enlarged to approximately 3 cm × 5 cm, involving most of the posterior tracheal wall, the left main bronchus, and the esophagus. On August 26, a gastrostomy with jejunal extension (PEG-PEJ) was created for enteral feeding, the ECMO circuit was exchanged, and empiric antifungal therapy with caspofungin and voriconazole was initiated while the differential diagnosis—including histiocytosis—was still being pursued. Definitive reconstruction was undertaken on August 28 while the patient remained on VV-ECMO.

Intraoperative findings via right posterolateral thoracotomy included a TEF exceeding 5 cm in length extending below the carina, near-complete destruction of the left main bronchus, extensive inflammatory involvement of the tracheobronchial bifurcation, and anterior esophageal perforation. No neoplastic tissue was identified. The operative field was heavily inflamed and contaminated, which precluded the use of prosthetic or pericardial materials.

**Figure 1:**
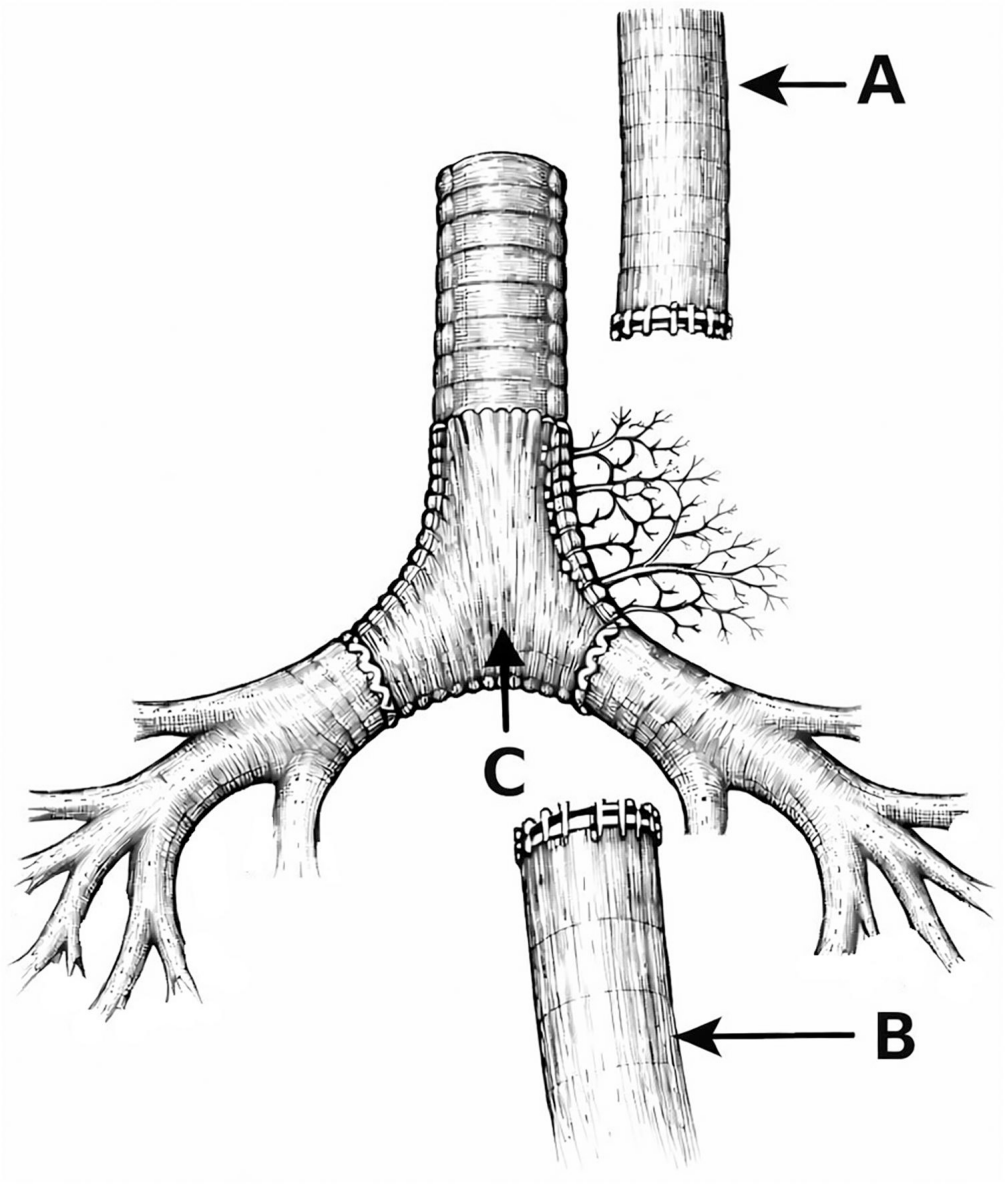
Schematic posterior view of the reconstructed airway segment. **(A)** Proximal esophageal stump; **(B)** distal esophageal stump; **(C)** vascularized esophageal muscular wall flap forming the reconstructed membranous carina and posterior walls of both main bronchi. Branching vascular arcade is visible on the right side of the flap. Suture lines indicate interrupted absorbable (PDS 4-0) fixation to residual tracheal and bronchial structures.

Esophageal continuity was deliberately sacrificed. The esophagus was transected and stapled proximal and distal to the diseased segment. A well-vascularized autologous esophageal muscular flap, measuring approximately 5 cm × 3 cm, was harvested from the remaining esophageal wall. The mucosal layer was completely stripped to expose the muscular layer, while the segmental blood supply from esophageal branches of the bronchial and intercostal arteries was carefully preserved. The muscular flap was rotated posteriorly and sutured to the residual tracheal and bronchial cartilaginous structures using interrupted absorbable sutures (PDS 4-0) to reconstitute the posterior wall of the carina and proximal main bronchi (Figure 1). The repair was tension-free throughout. Temporary bilateral bronchial stenting was achieved using Nelaton catheters introduced via the endotracheal tube. Alternative autologous tissue options were considered but deemed unsuitable: an omental flap would have required an additional laparotomy through a separate body cavity in a hemodynamically unstable patient on ECMO anticoagulation; intercostal muscle flaps were insufficient in size to cover a defect of this magnitude; pericardial tissue was avoided due to the extensive mediastinal contamination; and pleural flaps were precluded by inflammatory involvement of the ipsilateral pleura.

**Figure 2:**
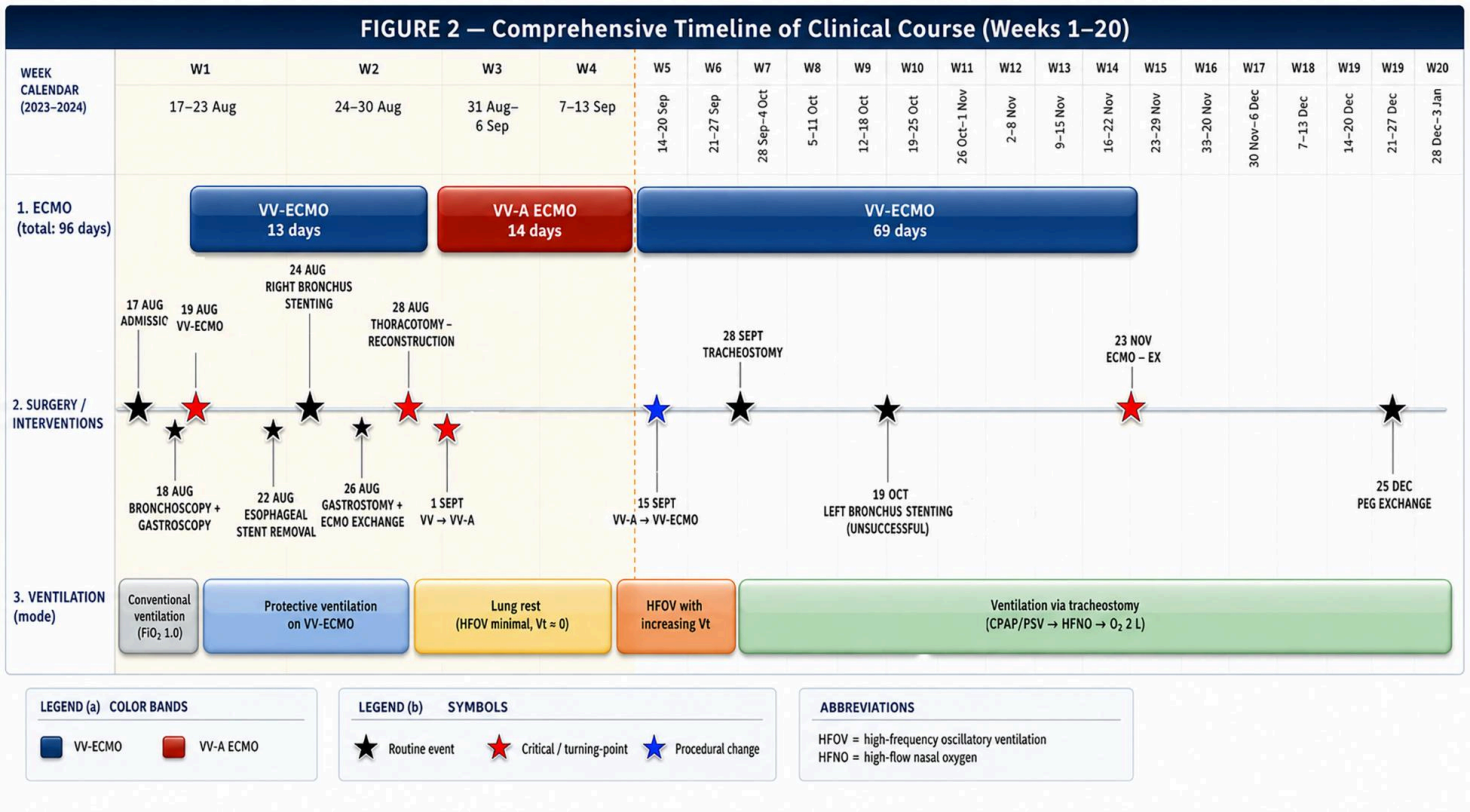
Comprehensive timeline of clinical course. Three lanes display parallel aspects of management from admission (August 17, 2023) through transfer to Leipzig (January 4, 2024). Lane 1: ECMO support (96 days) in three configurations—VV-ECMO (blue), VV-A ECMO (red) for right ventricular failure, and return to VV-ECMO. Lane 2: surgical and procedural interventions (red star = definitive airway reconstruction). Lane 3: ventilatory strategy progression from lung rest through tracheostomy-based weaning.

Histopathological examination demonstrated Aspergillus hyphae within the resected tissue, confirming invasive aspergillosis as the underlying etiology (9).

### Postoperative intensive care and ECMO course

Postoperatively, the lungs were intentionally kept almost unventilated for 14 days to minimize shear stress on the membranous reconstruction, with gas exchange maintained entirely by VV-ECMO. High-frequency oscillatory ventilation with very low tidal volumes was initially employed when ventilation was cautiously reintroduced, and prone positioning for 8–10 h daily was used to optimize oxygenation and dorsal lung recruitment.

Despite adequate gas exchange on VV-ECMO, chest radiography demonstrated near-complete bilateral opacification consistent with profound atelectasis. Serial transthoracic echocardiography (*n* = 16) was performed throughout the ECMO course to monitor ventricular function and pulmonary vascular resistance. Severe pulmonary hypertension and right ventricular failure developed; echocardiography on August 30 revealed severe right ventricular dilatation with significant tricuspid regurgitation and an estimated right ventricular systolic pressure of 100 mmHg, prompting emergent conversion to veno-arterial support on September 1 via mini-sternotomy: an 18 Fr EOPA Medtronic® arterial cannula was placed in the ascending aorta under echocardiographic guidance, converting the circuit to veno-veno-arterial (VV-A) configuration to stabilize hemodynamics, unload the failing right ventricle, and permit gradual reintroduction of ventilation. Over approximately 2 weeks of VV-A support, ventilation was carefully escalated with progressive increases in tidal volume, resulting in re-expansion of both lungs. Following normalization of right ventricular pressures confirmed echocardiographically, the aortic cannula was removed on September 15, the sternotomy was closed, and the patient was returned to VV-ECMO.

**Figure 3:**
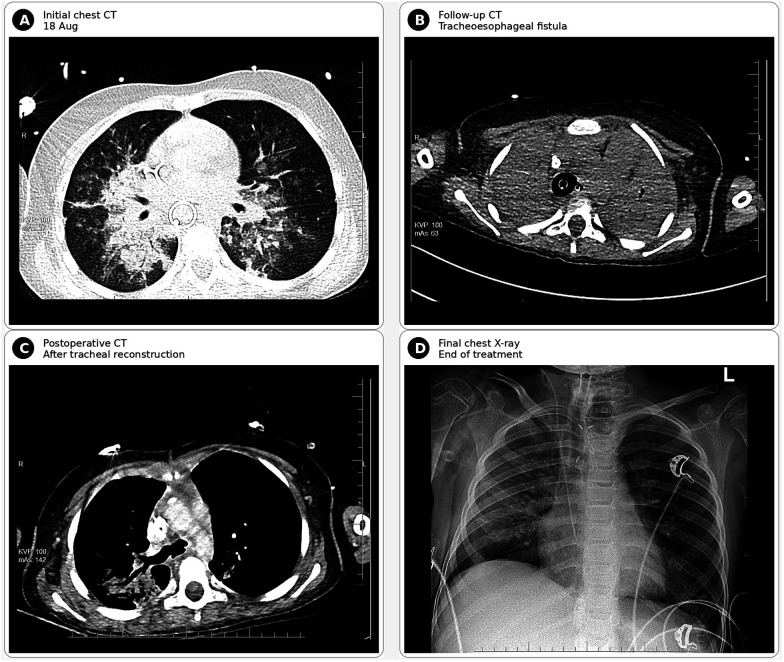
Serial imaging documenting the clinical course. **(A)** Contrast-enhanced chest computed tomography on hospital day 1 (August 18, 2023). Bilateral consolidation with predominantly perihilar distribution consistent with aspiration pneumonitis. A large, poorly defined soft-tissue density is visible in the posterior mediastinum, encasing the pulmonary arteries and compressing the left main bronchus. A covered esophageal stent is *in situ*; no definite tracheo-esophageal fistula is identified on this study. **(B)** Follow-up chest computed tomography demonstrating the tracheo-esophageal fistula with direct communication between the airway and the esophageal lumen at the carinal level, following resolution of the previously described mediastinal mass. **(C)** Postoperative chest computed tomography after tracheobronchial reconstruction with the esophageal muscular wall flap. The tracheal bifurcation is visible with patent origins of both main bronchi. Residual parenchymal changes and postoperative mediastinal alterations are present. **(D)** Posteroanterior chest radiograph obtained at the end of the intensive care stay (December 18, 2023), showing satisfactory bilateral lung expansion, tracheostomy cannula *in situ*, and percutaneous gastrostomy tube. No pneumothorax or significant pleural effusion.

VV-ECMO was continued to permit ongoing respiratory support and controlled ventilator weaning. A tracheostomy was performed on September 28, 2023, to facilitate reduction of sedation, neurological assessment, and awake ECMO management. A total of 73 bronchoscopic procedures were performed during the hospital stay for airway surveillance, bronchial toilet, and bronchoalveolar lavage (BAL). Regular bronchoscopic surveillance excluded anastomotic dehiscence. The tracheal reconstruction healed satisfactorily. The left main bronchus remained markedly narrowed, with features of malacia progressing to significant stenosis; two attempts at endoscopic stenting were unsuccessful due to distorted airway anatomy. No thromboembolic complications involving the central nervous system were observed during the entire ECMO course. Final decannulation from VV-ECMO was achieved on November 23, 2023, after a total of 96 days of extracorporeal support (Figure 3C).

### Anti-infective and supportive therapy

The diagnosis of invasive aspergillosis was established on histopathological examination of resected tissue, which demonstrated abundant Aspergillus hyphae (9). Serial galactomannan assays and fungal cultures from bronchoalveolar lavage fluid and blood were repeatedly performed throughout the hospital course but remained consistently negative. However, quantitative polymerase chain reaction (PCR) for Aspergillus DNA from peripheral blood returned positive, confirming systemic fungal invasion at the molecular level. These findings expose a well-recognized diagnostic gap: galactomannan and culture-based methods may fail in tissue-invasive aspergillosis, and molecular testing should be pursued alongside histopathological examination when clinical suspicion is high. The antifungal regimen was modified repeatedly over the course of treatment: empiric caspofungin and voriconazole were initiated preoperatively on August 26 while histiocytosis was still being considered in the differential diagnosis; following intraoperative histopathological confirmation of Aspergillus on August 28, voriconazole was replaced by liposomal amphotericin B (Ambisome); after approximately 6 weeks, amphotericin B was transitioned to voriconazole due to progressive hepatic dysfunction attributed to drug hepatotoxicity compounded by hepatic congestion from right ventricular failure; voriconazole was subsequently switched to isavuconazole in early November as hepatic dysfunction persisted; the isavuconazole dose was later escalated to 300 mg daily based on therapeutic drug monitoring. Nebulized amphotericin B (Fungizone) was administered intermittently as an adjunct. Antibacterial therapy was guided by serial microbiological cultures from blood, BAL fluid, and wound specimens, and included glycopeptides, oxazolidinones, and beta-lactam/beta-lactamase inhibitor combinations as indicated. Serial microbiological surveillance was performed at eight time points throughout the hospitalization, including blood cultures, BAL cultures, and fungal-specific assays. Serial chest radiography (*n* = 51) was performed throughout the ECMO course to monitor lung re-expansion and cannula position (Figure 2). Anticoagulation during ECMO was maintained with unfractionated heparin by continuous infusion, titrated to activated clotting time (target 160–200 s), at doses of approximately 40–100 U/kg/h. Heparin was withheld perioperatively and prior to bronchoscopic procedures. Supportive management included enteral nutrition via percutaneous endoscopic gastrostomy with jejunal extension (PEG-PEJ), continuous glucose monitoring with insulin therapy, and intensive respiratory and physical rehabilitation.

### Outcome

The patient improved gradually over the following weeks. He was discharged from intensive care on December 28, 2023 (Figure 3D), and transferred on January 4, 2024, to a specialized pediatric surgical center in Leipzig, Germany, for staged esophageal reconstruction. At the time of transfer, he required intermittent ventilatory support via tracheostomy, low-flow oxygen supplementation, and gastrostomy feeding. Neurological assessment confirmed satisfactory recovery, although long-term neurodevelopmental follow-up is ongoing. During subsequent management at the pediatric surgical center in Germany, a cervical salivary fistula was established to divert saliva, and the patient is currently being prepared for definitive esophageal reconstruction using an intestinal conduit. It should be noted that at the time of this report, esophageal continuity has not yet been restored, and the outcome remains interim pending completion of the staged reconstruction.

## Discussion

Inflammatory destruction involving both the trachea and esophagus is exceptionally rare in children and represents one of the most challenging scenarios in pediatric surgery. This case describes a massive acquired TEF with severe tracheobronchial injury caused by invasive mediastinal aspergillosis in a child without prior immunodeficiency. Although the patient was not classically immunocompromised, the concurrent diagnosis of new-onset type 1 diabetes mellitus with diabetic ketoacidosis may have contributed to transient immune dysfunction through hyperglycemia-induced impairment of neutrophil function and mucosal barrier integrity, potentially facilitating opportunistic fungal invasion. Aspergillus-related TEF has been reported predominantly in immunocompromised patients (6, 7), making the extent of necrosis and airway destruction in this case particularly unusual.

In this patient, early institution of VV-ECMO not only bridged the period of refractory respiratory failure but also permitted intentional lung rest following airway reconstruction—a strategy that, to our knowledge, has not been reported in the context of pediatric carinal surgery. When profound atelectasis led to severe pulmonary hypertension and right ventricular failure, temporary conversion to veno-arterial support allowed hemodynamic stabilization and gradual reintroduction of ventilation. Although the risk of complications rises with prolonged ECMO duration, in selected patients with otherwise unsurvivable pathology it may represent the only viable bridge to recovery (1–3, 10, 11).

The use of a vascularized esophageal muscular flap for posterior tracheobronchial reconstruction has not, to our knowledge, been previously described. The rationale for its selection was based on first principles: the esophageal wall offered immediate anatomical proximity to the defect, an intrinsic segmental blood supply from bronchial and intercostal arterial branches, sufficient surface area to cover the large posterior defect, and the inherent resistance of well-vascularized muscle tissue to infection (8). Among established autologous options, the omental flap has the strongest evidence base for use in infected mediastinal fields and has been successfully employed for tracheal reinforcement following resection, irradiation, or contamination (12). In the present case, however, the omental flap was not selected because it would have required an additional laparotomy in a hemodynamically unstable child on ECMO with systemic anticoagulation, adding substantial operative risk. The decision to sacrifice esophageal continuity represents a justified trade-off when airway survival takes absolute priority: the alimentary tract can be reconstructed electively once the inflammatory burden has resolved. In the present case, a cervical salivary fistula has since been established at the referring center, and the patient is currently undergoing preparation for definitive esophageal reconstruction using an intestinal conduit, an approach well described for long-gap esophageal replacement in children (13). This ongoing staged reconstruction supports the feasibility of the approach, although the final outcome remains to be determined.

Staged management was essential. Airway protection and reconstruction were prioritized, while restoration of esophageal continuity was deferred. This outcome would not have been possible without close collaboration between pediatric surgeons, cardiothoracic surgeons, intensivists, anesthesiologists, infectious disease specialists, endocrinologists, and specialized nursing and rehabilitation teams.

Invasive aspergillosis carries high mortality, particularly when diagnosis is delayed (14). In a series of 32 patients with invasive aspergillosis receiving ECMO support, overall mortality was 78%, with ECMO-related complications occurring in over half the cohort (15). The consistently negative galactomannan assays and fungal cultures in our patient, despite histopathologically and molecularly confirmed tissue invasion (positive Aspergillus DNA PCR from blood), demonstrate that conventional serological biomarkers alone cannot be relied upon to exclude invasive aspergillosis in this clinical setting. The antifungal management in this case required multiple sequential modifications driven by hepatotoxicity from amphotericin B and voriconazole, ultimately necessitating transition to isavuconazole with therapeutic drug monitoring, reflecting how difficult it is to maintain effective antifungal therapy in critically ill children on prolonged ECMO.

This report has several limitations. As a single case, the findings cannot be generalized, and the described approach required a highly specialized multidisciplinary setting with immediate access to ECMO, pediatric cardiac surgery, and advanced bronchoscopic capabilities—resources that are not universally available. The outcome remains interim: esophageal continuity has not yet been restored, and long-term airway patency, pulmonary function, and neurodevelopmental status require further follow-up. Patient selection for such an aggressive strategy must be carefully considered; children with pre-existing severe comorbidities, irreversible neurological injury, or uncontrolled multiorgan failure may not be appropriate candidates. Finally, the novelty of the esophageal muscular flap technique means that no comparative data exist, and further experience will be needed to confirm its applicability beyond this individual case.

## Conclusion

Massive acquired tracheo-esophageal fistula with extensive tracheobronchial destruction due to invasive aspergillosis is exceedingly rare in immunocompetent children. Prolonged ECMO support, used as an operative adjunct to enforce lung rest during reconstruction, combined with a vascularized esophageal muscular wall flap and close interdisciplinary care, made survival and functional recovery possible. Early recognition of atypical invasive infection and timely escalation to extracorporeal support may prove life-saving when airway destruction is otherwise irreparable.

## Patient perspective

*The following account was provided by the patient's mother approximately 18 months after discharge from our unit.*

“After transfer to Leipzig, our son spent several weeks in rehabilitation in Dresden. On May 24, 2024, he came home for the first time in nine months. He was prescribed a vibrating vest for airway clearance, which he uses daily and which has made a noticeable difference. In June he attended his kindergarten graduation ceremony with his classmates—every child had prepared a gift for him. I write this and I cry: it is an enormous joy simply to see my son standing there among his friends. He started school in August 2024 and is doing well, though I attend alongside him as his companion because he still needs support. He works with a physiotherapist, a speech therapist, and an occupational therapist. He walks on his own two legs, he speaks—his voice is quieter than normal but clearly understandable—and he breathes independently. He has gained weight and grown stronger. In October he was hospitalized in Leipzig with pneumonia and a pulmonary bacterial infection that required intravenous and inhaled antibiotics. The planned esophageal reconstruction has been postponed to allow his body to recover fully and overcome the infection. He turned eight years old in September 2024 and celebrated with family and friends. There is not a single day that we do not think of the team in Gdańsk. You are our family. Thank you for giving us our son back.”

## Data Availability

The original contributions presented in the study are included in the article/Supplementary Material, further inquiries can be directed to the corresponding author.
